# Recent Advances in Nanotechnology for Dendritic Cell-Based Immunotherapy

**DOI:** 10.3389/fphar.2020.00960

**Published:** 2020-06-30

**Authors:** Chen Qian, Li-Jun Yang, Hong Cui

**Affiliations:** Department of Pediatrics, Beijing Friendship Hospital, Capital Medical University, Beijing, China

**Keywords:** nanotechnology, dendritic cell, cancer, immunotherapy, antigen delivery, cross-presentation

## Abstract

Dendritic cells (DCs) are the most important antigen-presenting cells that determine cancer immune responses by regulating immune activation and tolerance, especially in the initiation stage of specific responses. Manipulation of DCs to enhance specific antitumor immune response is considered to be a powerful tool for tumor eradication. Nanotechnology, which can incorporate multifunction components and show spatiotemporal control properties, is of great interest and is widely investigated for its ability to improve immune response activity against cancer and even for prevention and avoiding recurrence. In this mini-review, we aim to provide a general view of DC-based immunotherapy, including that involving the promising nanotechnology. Particularly we discuss: (1) manipulation or engineering of DCs for adoptive vaccination, (2) employing DCs as a combination to more existing therapeutics in tumor treatment, and (3) direct modulation of DCs *in vivo* to enhance antigen presentation efficacy and priming T cells subsequently. We comprehensively discuss the updates on the application of nanotechnology in DC-based immunotherapy and provide some insights on the challenges and opportunities of DC-based immunotherapeutics, including the potential of nanotechnology, against cancers.

## Introduction

Cancer therapies have been evolving with advances in oncology (Couzin-Frankel, 2013). There has been some success with the use of traditional cancer therapies such as surgery (Krook et al., 1991; Wolf, 1991; Macdonald et al., 2001; Coffey et al., 2003), chemotherapy (Liu et al., 2011; Al-Lazikani et al., 2012; Koppelmans et al., 2012; Chen et al., 2017), and radiotherapy (Sauer et al., 2004; Abe et al., 2005). However, low-specificity, drug resistance, and side effects hinder the efficacies of these tumor treatments (Lippert et al., 2008; Guth et al., 2011; Housman et al., 2014; Rothermundt, 2015; Leary et al., 2018). Inducing or boosting up the patients’ own immune responses enable specific recognition and killing of tumor cells. Besides, immune response-mediated tumor eradication reduces the potential risks of toxicity and drug resistance (Beltran et al., 2011; Zhong et al., 2017). Therefore, in recent years, cancer immunotherapy is burgeoning and is considered to be the fourth important therapeutic method to deal with cancers. For example, the first tumor vaccine Sipuleucel-T was approved by the U.S. Food and Drug Administration (FDA) in 2010 for prostate cancer treatment (Higano et al., 2009; Kantoff et al., 2010). In the next year, the first immune-checkpoint inhibitor antibody Ipilimumab (anti-CTLA4 antibody) was approved for the treatment of advanced melanoma (Robert et al., 2011). In the next few years, multiple immune-checkpoint blockage antibodies were approved in various cancers and achieved great success (Brahmer et al., 2015; Le et al., 2015; Reck et al., 2016). Encouragingly, the Nobel prize in physiology and medicine honored James P. Allison and Tasuku Honjo for their discovery of the efficacy of inhibiting negative immune regulation in cancer therapy. Additionally, Tisagenlecleucel, the first chimeric antigen receptor-T cell-based adoptive transfer treatment for pediatric and young adult acute lymphoblastic leukemia and adult diffuse large B-cell lymphoma was approved in 2018 by the FDA (June et al., 2018; Maude et al., 2018).

Although great achievements have been made in cancer immunotherapy, only some percentage of patients can benefit from those promising immunotherapeutics, mainly owing to the immunosuppressive microenvironment of the solid tumor and immune tolerance to mono-therapeutics (Albini et al., 2018; Binnewies et al., 2018; Costa et al., 2018; Feng et al., 2018; Knudson et al., 2018; Zhao et al., 2018). Therefore, maximizing the potential and ability of the immune system to overcome tumors is critical (Zhang and Bevan, 2011; Nicholson, 2016). Dendritic cells (DCs) are the initiators of specific immune responses, and when activated effectively can lead to priming of T cells to elicit immune antitumor responses for tumor destruction (Rescigno et al., 1997; Leifer, 2017). More importantly, results of whole exome sequencing and RNA sequencing present the possibility of developing DC-targeting vaccines and DC-based immunotherapy (Leitner et al., 1999; Mathan et al., 2017). Manipulating DCs by taking advantage of the controllable and modifiable features of nanotechnology shows promising antitumor responses both in vitro and in vivo.

## DCs in Immune System

DCs are originated from dedicated hematopoietic precursor cells and transformed into DC in variable stimuli in physiological environment. (Thomas and Lipsky, 1996; Ardavin et al., 2001; Ardavin, 2003; Wang et al., 2019b). Circulating blood cells such as monocytes, white blood cells may differentiate to matured DCs owing to the crosstalk among diverse signals coordination (Rutella et al., 2006; Williams et al., 2013). DCs were demonstrated to play a crucial role in mediating innate and adaptive immune responses in 1970s by Ralph Steinman and Zanvil A. Cohn, who were honored with the Noble Prize for their discovery. Since then, DCs have been documented as the most effective antigen-presenting cells that activate primary and subsequent memory immune responses (Kadowaki, 2009).

**Figure 1:**
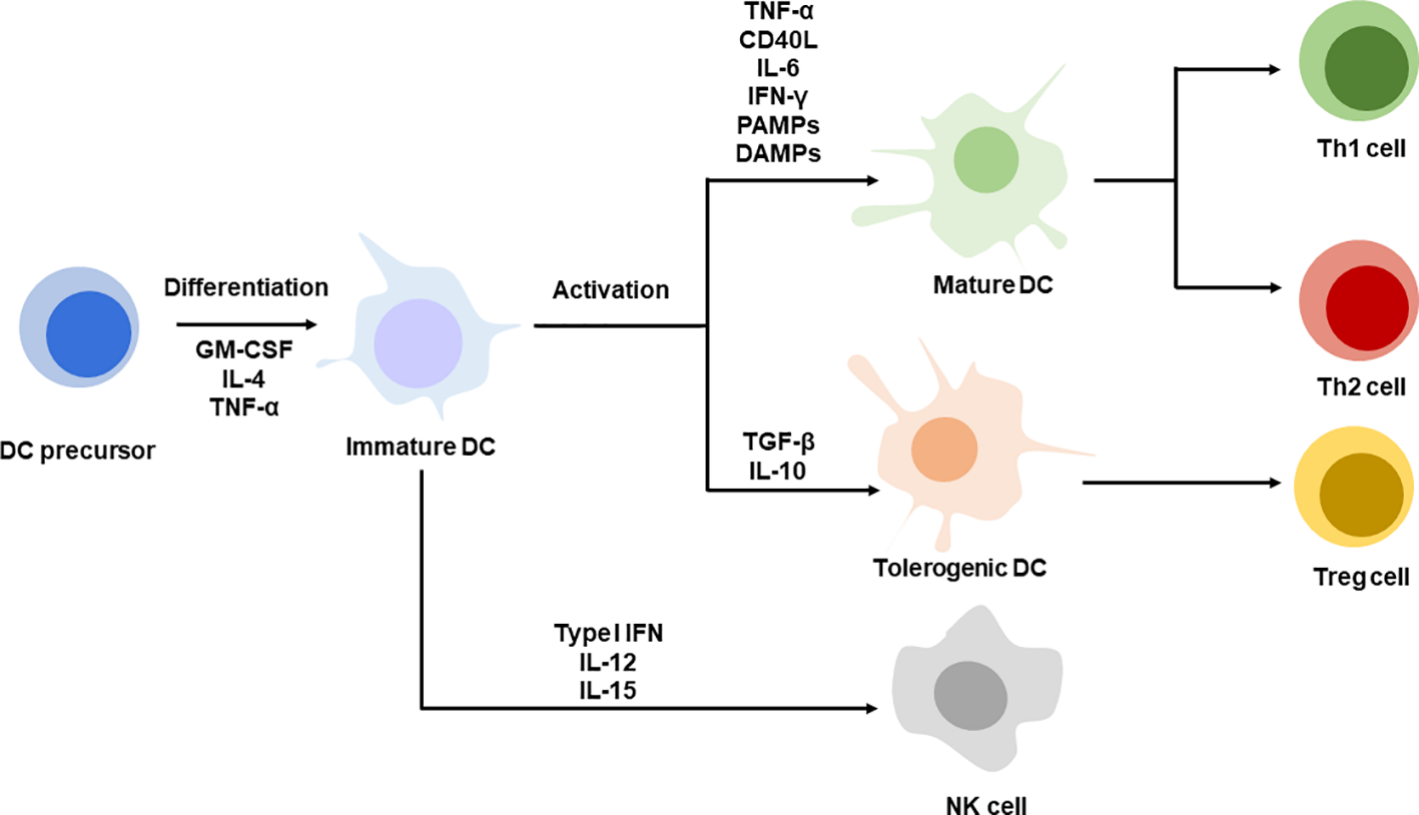
DC differentiation and maturation. DC precursors are differentiating into immature DC. Immature DCs are activated with specific stimuli, TNF-α, CD40L, IL-6, IFN-*γ*, PAMPs, DAMPs promote DC maturation, corresponding to Th1 or Th2 cells priming. DCs stimulate with TGF-*β*, IL-10 present tolerogenic phenotype leading to Treg differentiation. Immature DC decorated with type I IFN, IL-12 and IL-15 favor NK cell function.

DCs as antigen-presenting cells encounter antigens and present them to T cell for priming in the form of antigen peptide-major histocompatibility complex (MHC) (Cohen et al., 2003; Zehn et al., 2004). Antigens may circulate through peripheral blood and stimulate DCs or captured by tissue resident DCs such as lymph node, spleen. Whatever, DCs’ received antigens are required to migrate to the lymphoid organs which determines the subsequent T cell activation efficacy due to the abundance of T cells in the lymphoid organs (Mempel et al., 2004) (Segura and Villadangos, 2009). The success of DCs’ activation requires two signals, antigens and activation stimuli. The second activation signals may be provided exogenously such as by lipopolysaccharide (Morrison and Kline, 1977), Toll-like receptor (TLR) ligands (Kawai and Akira, 2007), and antibodies targeting activation of receptors like tumor necrosis factor (Schnurr et al., 2000), proinflammatory cytokines IFN-*γ*, *etc* (Simmons et al., 2012; Minton, 2014). Endogenous damage-associated molecular patterns such as high mobility group proteins (Raucci et al., 2007), calreticulin (Li et al., 2015), and heat shock proteins with immunogenic features are also capable of maturing DCs (Bethke et al., 2002). Similarly, two signals are required for T cell priming; one is a specific antigen-MHC I/II complex and the other one is a costimulatory signal expressed on activated DCs. mDCs with antigen-MHC complex expression induce T cells to differentiate into Th1 or Th2 cells under the condition of variable cytokines, for example, IL-12, which is essential to activate cytotoxic T cells. In some other cases, under the condition of IL-4, it may switch immunity with antibody secretion. Mostly, these activated CD8+ T cells are fully functional, with cytotoxicity and ability to secrete IFN-*γ* for highly effective and specific killing of cancer cells. Other than cytotoxic T cell stimulation, matured DCs (mDCs) may also function to neutralize antibody secretion by B cells in the way of IL-10 and IL-33 secretion, driving Th2 immunity and influencing immunoglobulin subtype polarization (Segura et al., 2013). Additionally, DCs with inflammatory features induce Th17 differentiation, which promotes cytotoxic T cell responses and regresses tumor (Murugaiyan and Saha, 2009; Segura et al., 2013; Guéry and Hugues, 2015). Apart from stimulating specific adaptive immunity, mDCs can also be decorated with IL-12, IL-15, and type I IFNs which are positive to NK cell functions in innate immunity. Thus, the DCs act as a bridge between innate and adaptive immunity for host defenses (**Figure 1**). As discussed above, the immature DCs that are usually found in peripheral lymphoid tissues process antigens without activation stimuli and are capable of presenting antigen-MHC to naïve T cells, resulting in tolerance to T cell responses (Probst et al., 2005).

## Manipulation of DCs for Vaccination

DCs that initiate adaptive immune responses, including antitumorigenesis, are of great interest worldwide, owing to their ability to be able to present tumor-associated antigens and prime subsequent antitumor responses. Because of their critical role with professional presentation expertise in the immune system, exploiting DC for vaccination is considered to provide a powerful tool to prevent and cure infections and cancers (Lizotte et al., 2016; Roden and Stern, 2018; Wang et al., 2019a; Wen et al., 2019). As initiators of adaptive immunity, DCs are ideal targets for *ex vivo* education and adoptive vaccination, which can induce specific antitumor immune responses in patients *in vivo* (Lövgren et al., 2018). Conventional techniques to develop DC-based vaccines involved isolation or culture precursor cells from patients’ peripheral blood, loading them with antigens *in vitro* and applying certain maturation stimuli to promote DC maturation. Precursor cells such as CD34+ hematopoietic cells, monocytes cultured with certain stimuli enabled differentiating into immature DCs. In order to differentiate and expand DCs *in vitro*, immature DCs are loaded with tumor antigens such as tumor lysates, peptides, proteins, nucleotides, or fused with tumor cells, under the condition of stimulatory molecules. Challenges remained although more techniques have been developed. Peptides can be loaded directly on MHC molecules; however, it requires clear and definite information not only on the epitopes such as sequence and conserved motif, but also on an individual’s HLA configuration that determines the type of immune responses and adaptive immunity. Clinical study is ongoing to confirm the immunogenicity of peptide-loaded DC vaccination (NCT02334735). Instead of peptides, loading of proteins or lysates has also been used for numerous cancer treatments (NCT00045968). Although these require further intracellular processing, the major advantage of using whole protein processing is its potential to induce both CD4+ and CD8+ T cell responses (Constantino et al., 2016; Wang et al., 2017; Sharma et al., 2018). To realize the DC function of initiating specific antitumor immunity, DCs loaded with tumor-associated antigens require stimuli (example, CD40L or TLR agonist) for maturation. The mDCs are then transferred back to the patients to mediate specific immune response. The whole process is complicated and requires skilled operation and high cost. Most clinical studies on DC vaccines are in combination with chemotherapy (NCT03688178, NCT03657966, NCT03047525) (Kongsted et al., 2017), radiotherapy (NCT03226236,), targeted agents (Ogasawara et al., 2018; Palma et al., 2018), or immunotherapeutic regimens (NCT03546426, NCT03735290, NCT03450044, NCT03735589). Clinical studies implied that technologies that combine antigen loading and various combination agents are required and of great potential.

Nanotechnology refers to the technology utilizing nanosized materials that range from 10 to 100 nm in size; these are emerging as an ideal tool for cancer therapy. Nanomaterials with divergent compositions enable passive or active targeting, which enhances the efficacy and reduces the toxicity of treatment (Liu et al., 2018; Wang and Mooney, 2018; Feng et al., 2019). The ability of DCs to capture, process, and present antigen to induce T cell priming is essential in adaptive antitumor immunity. Therefore, nanomaterials targeting DCs are considered to be a promising tool to boost an efficient and specific anticancer immune response. Nanomaterials also incorporate multifunctional molecules together to modulate part or whole of the antigen presentation (Parvanian et al., 2017; Sangtani et al., 2017). Functional nanomaterials with lysosome escaping ability have been widely reported; these nanomaterials are favorable for antigen delivery to the cytoplasm and their intracellular digestion, which further contributes to antigen processing and presentation (Jiang et al., 2018; Zhong et al., 2019). Lipid-based nanoparticles and polymeric nanoparticles (Reichmuth et al., 2016; Zhou et al., 2016; Gulla et al., 2019; Wen et al., 2019; Lin et al., 2020), with better biocompatibility, have been reported in the development of DC targeted vaccines for anticancer and antiviral treatments. Liposome-based nanoparticles containing molecules such as trivalent influenza antigen, OVA, and heat shock proteins, have been investigated for their ability to activate the immune system in disease treatment. Delivery of protein, peptide, and nucleic acid antigens, which can induce corresponding immune responses, to DCs *via* nanomaterials might probably increase their circulation and reduce their degradation *in vivo*, which are necessary for future approaches that aim to directly activate DCs *in vivo* (Rogel et al., 1985; Paglia et al., 1996; Ghadersohi and Sood, 2001; Goodwin and Huang, 2017; Wongso et al., 2017; Dellacherie et al., 2018). Moreover, nanomaterials (*e.g.*, gold, aluminum nanoparticles) probably enhance internalization at the single cell level (Wang et al., 2015; Li et al., 2016; Wang et al., 2016). Aluminum, which was documented as an adjuvant for Th2 stimulation, failed to generate CD8+ T cell responses (Ho et al., 2018). However, using nanosized aluminum particles (100 nm) stabilized by PEG-containing polymer showed higher internalization in antigen-presenting cells and activated CD8+ T cells for curing cancer. Thus, nanotechnology not only improves the bio- or physiochemical features of antigen formulation through antigen protection, quantity improvement, function combination, and antigen presentation pathway, but also redefines the efficacy of traditional agents or materials with potential novel functions in antitumor immunity modulation (Zupančič et al., 2017; Smith et al., 2018; Zhong et al., 2019), which are all beneficial for the subsequent adaptive immunity against cancers. Importantly, a few cases that used nanotechnology for DC vaccination have been reported. For instance, liposome based Tecemotide, AS15, DepoVax, decorating with tumor-associated antigens are in clinical trials (Berinstein et al., 2012; Butts et al., 2014; Vansteenkiste et al., 2014). Virus-like nanoparticles CYT004-MelQbG10 delivering melanoma-associated antigen peptide are in Phase II clinical trials (Speiser et al., 2010). These pre/clinical trials evidenced the potential targeting DC for antitumor immunotherapy.

## *In Vivo* Activation of DC for Enhancing Antigen Presentation

**Figure 2:**
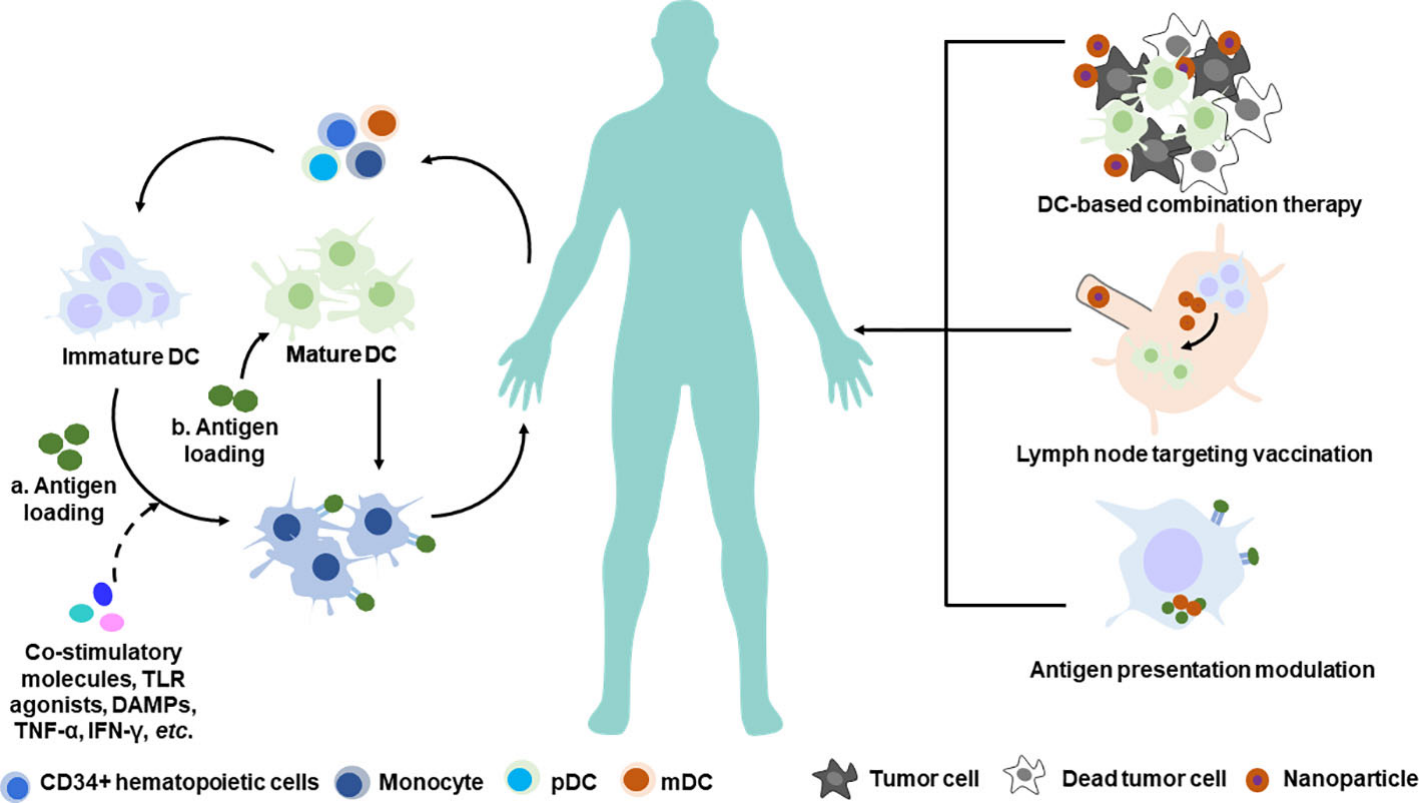
Nanotechnology-based manipulation of DCs therapy. Conventional strategy of DC-based adoptive transfer therapy (left) and novel strategy employing nanotechnology for future DC-based immunotherapy (right). a. Antigen loading on immature DC in the scenario of DC maturation stimuli. b. Antigen loading on mature DC directly. pDC, plasmacytoid dendritic cell; mDC, myeloid dendritic cell.

The traditional approach of activating DCs *in vitro* and transferring them back into patients is expensive, labor-dependent, and difficult to evaluate antitumor immunity generation parallelly in patients. Targeting DCs for *in vivo* activation with antitumor immunity enhancement is another promising DC-based therapeutic strategy to induce specific antitumor immune responses; this approach directly activates DCs *in vivo* and can potentially generate a large number of antitumor responses. Nanotechnology-enabled spatiotemporal delivery of formulations aimed to directly activate DCs has been demonstrated in numerous cases (**Figure 2**). These formulations can be classified into three categories: (1) those that target and activate local DC response in the tumor microenvironment in addition to chemotherapy/radiotherapy (Sau et al., 2018); (2) those that target and activate lymph node-resident DCs, and in addition, target activation receptors on DCs, thereby boosting specific T cell response (De Koker et al., 2016; Jiang et al., 2017); and (3) those that modulate intracellular antigen presentation process for higher cross-presentation efficacy (Sil et al., 2019; Wang et al., 2019a).

### DC-Based Combination Therapy

Because of the heterogeneous features of variable tumors and their immune microenvironment, “cold” tumors with low immunogenicity show enrichment of immunosuppressive cells such as macrophages and myeloid-derived suppressor cells around the microenvironment to escape from the immune surveillance (Smyth et al., 2006; Dadi et al., 2016; Lussier and Schreiber, 2016; Haanen, 2017). Therefore, combination therapies aimed to change the tumor immunogenic phenotype provide a means to reduce the mortality associated with cancers (Da Silva et al., 2016; Nam et al., 2019). Chemo-immunotherapy approach that incorporates doxorubicin (DOX) and indoximod (IND) with phospholipid as a prodrug shows improved pharmacokinetics and accumulation in 4T1 breast tumor model. In such an approach, the chemo drugs induce tumor cell death, eliciting immunogenic reactions along with the debris being taken up by DCs, leading to further antigen presentation and naïve T cell priming. The activated T cells present cytotoxic effects on tumor cells through perforin and release of IFN-*γ* for robust killing of both primary and metastatic tumors. Not only does the dual-functional liposomes exert synergistic antitumor effects *via* DOX and IND components, but also the immune-checkpoint blockage combination treatment which further boosts immune responses allowing metastatic tumor eradication (Johnson et al., 2017; Lu et al., 2018). Radiotherapy is also known to promote CD8+ T cells around the tumor, however, radiation-induced immunosuppression may lead to treatment failure. Bismuth sulfide nanoparticles that conjugate immunoactive polysaccharide can increase radiotherapy sensitivity and activate DCs. Meanwhile, the nanoparticle can further enhance DC maturation and their distribution in tumor. Several studies have indicated that photodynamic therapy, which induces tumor cell lysates, is usually accompanied with damage-associated molecular pattern release that promotes DC activation, leading to specific antitumor immunity. Formulating photosensitizer is a major challenge in photodynamic therapy and further acceptance in clinic. The application of nanotechnology is a stride forward in solving this problem to some extent (Lucky et al., 2015), which favors photodynamic and DC combination therapy (Chen et al., 2016; Yu et al., 2019).

### Lymph Node Targeting Vaccination

Peripheral DCs that encounter antigens are required to be trafficked to the lymph node where abundant lymphocytes are located and enabled highly efficient T cell priming. Moreover, diverse cytokines in the lymph node further promote DC maturation and exert function (Manfredi et al., 2008; Martín-Fontecha et al., 2009; Granot et al., 2017). Modifying antigens to target the lymph node and enhance lymph node-resident DC uptake was demonstrated to be a promising strategy in vaccine designing to initiate effective immune responses (Van Herck et al., 2018). Developing delivery systems with variable size can greatly influence the lymph node trafficking and DC uptake of antigens (Bachmann and Jennings, 2010; Kuai et al., 2017; Zhang et al., 2018). Proteins or subunit viral antigens within 10 nm are mostly incorporated with adjuvants to form larger-sized particles or aggregates. Supramolecular antigen formulations such as virus-like particles range from 20 to 200 nm. Virosomes with liposomes and antigens are approximately 100–200 nm (Jennings and Bachmann, 2007; Bachmann and Jennings, 2010). These antigen formulations are presented in the form of nano- or microparticles. Zhang et al. (2018) systemically investigated the influence of size and charge on the micelle-like formulations and their contributions to modulating immune responses, including lymph node accumulation, and DC internalization and immunogenicity, which provide an insight into the micelles and the generated immune responses. Lymph node accumulation and interaction properties of antigen-presenting cells are the two most important factors that influence the immunogenicity of nanoparticulated/nanoformulated antigens. Lymph node draining and DC uptake capability are both size-dependent processes, with a diameter of 10–200 nm considered to be optimal to stimulate immune response. Additionally, the authors claimed that the optimized size range was relatively independent of the materials, as the efficacy of lymph node draining and DC internalization is critically attributed to the particle size. However, variable materials with surface charges might influence the internalization capability of the antigen-presenting cells. Micelles with positive charged surface are beneficial for the enhancement of host antibody production (Reddy et al., 2007; Irvine et al., 2013). The previously published reviews provide an overview of materials on physiochemistry properties of antigen formulation in exerting effective immune responses.

Zhang et al. (2019) designed and synthesized a hybrid nanoparticle including the TLR 7/8 agonist imiquimod, TLR4 agonist monophosphoryl lipid A, polycaprolactone–polyethylene–polycaprolactone copolymer, dioleoyl-3-trimethylammonium propane, and 1,2-Distearoyl-sn-Glycero-3-Phosphoethanolamine-mannos. The hybrid nanoparticles enabled trafficking to secondary lymphoid tissues, spatiotemporal delivery and stimulation of both extracellular and intracellular TLRs, which was critical for efficient DC activation. It was demonstrated that the hybrid nanoparticles favorably enhanced the DC uptake of tumor antigen and cytokine secretion by mDCs. Additionally, homogenous tumor cells re-inoculated into C57BL/6 mice were inhibited upon treatment with the hybrid nanoparticles, highlighting the promising specific antitumor immune responses mediated by the nanoparticles.

### Modulating Antigen Presentation for DC Vaccination

Antigen processing is an essential process for the final antigen presentation efficacy followed by lymph node draining accumulation and uptake by antigen-presenting cells. Thus, regulating intracellular antigen processing is important for antigen presentation and subsequent T cell priming. DCs are considered with well capacity for lysosomal proteolysis. It was documented that macrophages containing abundant lysosomal proteases enabled internalized protein degradation rapidly. In contrast, DCs with limited lysosomal capacity due to poor proteolysis degraded antigens slowly *in vivo* thus preserved antigen for an extended period. The limited proteolysis capacity benefits antigen presentation (Delamarre et al., 2005). Moderate lysosomal capacity of DCs favored antigen presentation implied us the opportunity of other pathway modulation in enhancing antigen degradation and presentation. It has been reported that inducing autophagy, an intracellular degradation and clearance process of unnecessary or dysfunctional components, is beneficial for antigen processing by DCs and leads to highly effective T cell stimulation. Wang et. al. combined a model antigen peptide OVA_257–264_, autophagy inducing peptide Beclin1, and a pH sensitive polymer together by covalent conjugation and fabricated an autophagy-inducing nanoactivator, which could induce autophagy in DCs, and evaluated the antigen-specific immune responses both *in vitro* and *in vivo* (Wang et al., 2015; Wang et al., 2019a). They demonstrated that induction of autophagy improved the antigen-presenting efficacy and T cell activation. Notably, the MHC I-OVA_257–264_ complex expression was significantly decreased on combining the nanoactivator with an autophagy inhibitor. Furthermore, cross-presentation DCs and antigen-specific T cells increased, along with the appearance of specific antitumor immune responses, tumor infiltrated cytotoxic T cell accumulation, and tumor growth inhibition, indicating that the nanoactivators were capable of initiating antigen-specific immune responses *in vivo* for the eradication of established solid tumors.

## Conclusion

Considering that DCs play a vital role in priming naïve T cell and inducing adaptive immunity, tremendous efforts have been made in manipulating DC for cancer immunotherapy. It is documented that adoptive DC transfer can elicit specific immune responses in approximately 70% patients (Draube et al., 2011). Controlled parallel studies and comparative analysis are limited but essential for outcome evaluation and further clinical translation of adoptive DC vaccination. Moreover, immunosuppressive patients and intrinsic tumor-associated factors such as inhibitory ligands and Tregs do not favorably respond to DC-based immunotherapy. Hence, there is a need to improve therapeutic efficacy by manipulating DCs independently in the primary step. Developing combination strategies are expected to be promising in the exploitation of DC vaccines (Kalinski et al., 2013). Taking the advantages of nanotechnology, manipulation, and engineering of DCs *in vitro* can be simplified and its activation efficiency can be improved, which could increase the therapeutic efficacy of adoptive DC transfer treatment. In addition, nano-engineering can allow *in vivo* DC activation, mimicking natural antigen presentation followed by priming processes. Directly activating DCs *in vivo* fully employed the moderate lysosomal proteolysis feature that degraded antigens slowly which enabled sustaining antigen presentation. Making use of nanotechnology can also allow the conjugation of DCs to extraordinary functional molecules, which can further enhance antigen presentation, mediating sophisticated crosstalk inside the antigen-presenting cells and or work together with other cells, thereby contributing to the efficacy of DC-based immunotherapy. Another challenge to conventional DC-based therapeutics is that activated DCs are required to migrate to lymph nodes and prime T cells for specific antitumor responses; however, the majority of the DCs are unable to reach the lymph nodes, leading to limited specific immune responses. In contrast, nanoparticles enable higher lymph node accumulation, contributing to highly effective lymph node draining. Emerging nano- and bio-engineering techniques that fabricate lymphoid organs are promising to provide an artificial environment for DCs and T cell priming. Thus, nanotechnology is a promising approach for DC-based therapeutics and can promote multifield development with great impact on clinical cancer treatment.

## Author Contributions****

CQ, L-JY, and HC wrote the manuscript. HC provided advice both on the review writing and discussion.

## Funding

This work was supported by Beijing Municipal Science & Technology Commission (Z171100001717020).

## Conflict of Interest

The authors declare that the research was conducted in the absence of any commercial or financial relationships that could be construed as a potential conflict of interest.
